# Effects of Amino-Functionalized Carbon Nanotubes on the Crystal Structure and Thermal Properties of Polyacrylonitrile Homopolymer Microspheres

**DOI:** 10.3390/polym9080332

**Published:** 2017-08-02

**Authors:** Hailong Zhang, Ling Quan, Lianghua Xu

**Affiliations:** 1 School of Civil Engineering and Communication, North China University of Water Resources and Electric Power, Zhengzhou 450045, China; 2 Key Laboratory of Carbon Fiber and Functional Polymers Ministry of Education, Beijing University of Chemical Technology, Beijing 100029, China; xulh@mail.buct.edu.cn; 3 School of Electric Power, North China University of Water Resources and Electric Power, Zhengzhou 450045, China; quanling@ncwu.edu.cn

**Keywords:** polyacrylonitrile, amino functionalized CNTs, microspheres, in situ polymerization

## Abstract

Amino-functionalized multi-walled carbon nanotube (amino-CNT)/polyacrylonitrile (PAN) microspheres with diameter of about 300–400 nm were prepared by in situ polymerization under aqueous solution. The morphology, crystal structure, and thermal properties of amino-CNTs on a PAN homopolymer were investigated by scanning electron microscopy, transmission electron microscopy, Fourier transform infrared spectra, X-ray diffraction, and differential scanning calorimetry. The results showed that the amino-CNTs had a significant influence on the morphology of microspheres, and the PAN matrix were grafted onto the surface of amino-CNTs with interfacial bonding between them. The XRD studies showed that the crystal size of amino-CNT/PAN microspheres with lower crystallinity was bigger than in the control PAN homopolymer. The analysis of thermal properties indicated that the amino-CNT/PAN microspheres with lower glass transition temperature had a lower initial temperature and velocity of evolving heat during the exothermic processing as compared with the PAN homopolymer. These results suggested that the incorporation of amino-CNTs into the PAN homopolymer matrix was beneficial for controlling the heat released during the stabilization processing.

## 1. Introduction

Due to their unique structure and exceptional properties, such as high mechanical strength and excellent electrical and thermal properties, carbon nanotubes (CNTs) have received much attention from researchers in various fields [1]. However, the homogeneous dispersion of CNTs in a polymer matrix remains a challenge owing to the strong van der Waals forces between the tubes and the large aspect ratio. Moreover, the poor interfacial bonding between CNTs and the polymer matrix limits the full potential properties of CNTs as the reinforcements. So, various methods have been studied to solve these problems, such as non-covalent and covalent modification [2,3]. Non-covalent modification does not change the structure of CNTs, but it is hard to maintain the homogeneous dispersion and improve the interfacial interaction. Therefore, covalent modification is considered to be an effective method to obtain the most homogeneous dispersion and the better interfacial bonding between the CNTs and polymer matrix [4].

Polyacrylonitrile (PAN) is an important polymer generally used in the carbon fiber field due to its excellent mechanical and thermal properties. For the PAN polymer, thermal stabilization is one of the most important processes, which contain many reactions including cyclization, dehydrogenation, and oxidation in different atmospheres [5,6]. Ouyang et al. [7] reported the effect of itaconic acid (IA) on the structural evolution and thermal behavior through Fourier transform infrared spectroscopy (FTIR) and differential scanning calorimetry (DSC) analysis. Zhao et al. [8] studied the evolution of structure during heat treatment of PAN fiber under air atmosphere, and suggested that the addition of IA relaxed exothermic reactions. Zhang et al. [9] reported that the CNTs, after modification by hydrogen peroxide, increased the value of the heat flow for the PAN copolymer in a nitrogen atmosphere. Small functional groups, such as amino-groups, have been used by many researchers to graft onto the PAN homopolymer macromolecule chains in order to change the thermal stabilization processing [10,11].

The incorporation of CNTs into PAN to obtain composite fibers with remarkable mechanical and thermal properties has been studied by many researchers [12,13,14,15,16,17]. To obtain a homogeneous dispersion of CNTs in the PAN matrix as well as a strong interaction between the CNTs and PAN polymer, the CNTs were often modified by strong acid, and the CNT/PAN composites were prepared by solution blending. However, solution blending requires the removal of a large amount of solvent that pollutes the environment and consumes a lot of energy, and this method does not form strong interfacial bonding between CNTs and the polymer matrix. In recent years, in situ polymerization has been proposed as an effective way for the fabrication of CNT/polymer composite materials. Moreover, composites prepared by the in situ polymerization method show relatively good properties with a very low CNT content [18,19]. CNT/polyimide composites with a homogeneous dispersion of CNTs have been obtained by in situ polymerization, which showed that the CNTs participated in the reaction through the formation of amino bands [20,21]. For the PAN polymer, an acrylonitrile (AN) monomer with a nitrile group was used for grafting; for example, AN and aniline formed a chemical bond through the reaction between the nitrile group and amino group [22]. Therefore, the amino group on the surface of CNTs may also react with the nitrile groups in the PAN macromolecular chains via in situ polymerization.

In this study, the in situ polymerization technique under aqueous solvent was used to prepare amino-functionalized multi-walled carbon nanotube (amino-CNT)/PAN homopolymer microspheres. The dispersion of amino-CNTs and the interfacial bonding between amino-CNTs and the PAN homopolymer were investigated. Moreover, the morphology and crystal structure of microspheres were characterized. Finally, the thermal properties of amino-CNTs in the PAN homopolymer matrix were studied. 

## 2. Experimental and Methods

### 2.1. Materials and Amino-CNTs

Multi-walled carbon nanotubes (CNTs) were obtained from Shenzhen Nanotech Port Co. Ltd. (Shenzhen, China), and were synthesized by the chemical vapor deposition method with a CNT content with a purity of more than 95%. The length and the diameter of the CNTs was about 1–10 μm and 10–15 nm, respectively. The amino-functionalized carbon nanotubes (amino-CNTs) were obtained by the chemical modification method, which is outlined in a previous publication [10]. Acrylonitrile (AN) of analytical grade was procured from Beijing Xingjin Chemical Factory (Beijing, China) and distilled to remove any inhibitors before application. Hydrochloric acid (HCl), hydrogen peroxide (H_2_O_2_), thionyl chloride (SOCl_2_), tetrahydrofuran (THF), and triethylenetetramine (TETA), all of analytical grade, were obtained from Sinopharm Chemical Reagent Co. Ltd (Shanghai, China), and all other solvents were used without further purification.

Pristine CNTs were suspended in 3 mol HCl with a concentration of 1/50, and were sonicated for 1 h to remove the metallic catalysts. Then the mixture was rinsed with deionized water by filtration through a 2.0 μm polytetrafluoroethylene (PTFE) membrane until the potential of hydrogen of rinsing was about 7. Finally, the solid was dried in a vacuum oven for 12 h at 60 °C. The purified CNTs were dispersed in 30% H_2_O_2_ solution in an ultrasonic bath for 2 h, and then stirred for 24 h at 70 °C to obtain the COOH-CNTs (acid-CNTs). The mixture was filtered through the 2.0 μm PTFE membrane and washed with deionized water. The filtered solid was dried for 12 h at 60 °C. Then, the acid-CNTs were suspended in SOCl_2_ with an ultrasonicator for 2 h at room temperature and stirred for 24 h at 75 °C. The suspension was filtered through the 2.0 μm PTFE membrane and washed with anhydrous THF, and then dried in a vacuum oven at room temperature for 1 h. The COCL-CNTs powders were immediately added into excessive TETA, sonicated for 2 h, and stirred for 24 h at 120 °C to obtain the amino-CNTs. The mixture was filtered and washed with anhydrous ethanol, then dried in the vacuum oven for 12 h at 60 °C. 

### 2.2. Fabrication of Amino-CNT/PAN Microspheres 

Amino-CNT/PAN microspheres were prepared by aqueous phase precipitation in situ polymerization. The amino-CNTs and deionized water were put into a 250-mL three-necked flask under an ultrasonic bath for 30 min. Then, the AN monomer was charged into the three-necked flask and sonicated with mechanical stirring for 30 min at 50 °C under a nitrogen atmosphere. The initiator was added and the mixtures were allowed to polymerize for 2 h, after which the heat was reduced to room temperature. The mixture was filtered using a 2.0-μm PTFE membrane under vacuum and washed with ethanol to remove unreacted AN monomer, and then dried in a vacuum oven at 45 °C for 12 h. The quantity of CNTs in microspheres was 1% by mass. The control PAN homopolymer was polymerized under the same conditions without amino-CNTs.

### 2.3. Characterization

Fourier transform infrared (FTIR) spectrum (Nicolet 5700 spectrometer, Thermo Nicolet Company, Madison, WI, USA) was recorded for samples pressed into a pellet with KBr and scanned in the range from 400 to 4000 cm^−1^ at 4 cm^−1^ resolution. Scanning electron microscopy (SEM) (Hitachi, Tokyo, Japan) with a S 4700 instrument at 20 kV was acquired to examine the morphology of CNT/PAN. The powder samples were coated with a thin platinum layer by ion beam coater. Transmission electron microscopy (TEM) (Hitachi, Tokyo, Japan) was used to characterize the morphology of the modified CNTs and the PAN matrix grafted onto the surface on the amino-CNTs on a Hitachi H-800 electron microscope with an operating voltage of 200 kV. The powder samples were dispersed into alcohol solvent by ultrasonic bath at 40 kHz for 5 min, and then dripped onto a copper grid covered with a perforated carbon film. DSC curves were carried out in the different atmospheres with heating rates 10 °C min^−1^ by a Q 100 instrument (TA Company, Boston, PA, USA), and the glass transition temperature was measured by DSC with heating rate of 60 °C min^−1^ under a nitrogen atmosphere. The X-ray diffraction (XRD) (Rigaku, Japan) experiments of the powder samples were carried out using a D/max 2500VB2+/PC diffractometer with Cu Kα radiation at 30 kV and 20 mA, which was in the angle range of 2θ = 5~55° with the scan speed of 10°/min and a step of 1.0 s. The average crystal size (*L_c_*) was determined from the strong diffraction peak centered at 2θ ≈ 17° using the Scherrer equation [23]:

(1) $$Lc=\frac{K\lambda }{\beta \cos\theta }$$

where *K* is the Scherrer parameter that was taken to be 0.89; λ = 1.54056 nm is the wavelength of the radiation; and β is the breadth at half maximum intensity in radians at 2θ ≈ 17°.

Crystallinity was calculated from the XRD curves of the powder samples according to the following formula [24]:

(2) $$C(\%)=\frac{A_{c}}{A_{a}+A_{c}}$$

where *A_c_* is the integral area of the crystalline zone around 2θ ≈ 17°; and *A_a_* is the integral area of the amorphous zone. 

## 3. Results and Discussion

### 3.1. Morphology of the Amino-CNT/PAN Microspheres

**Figure 1:**
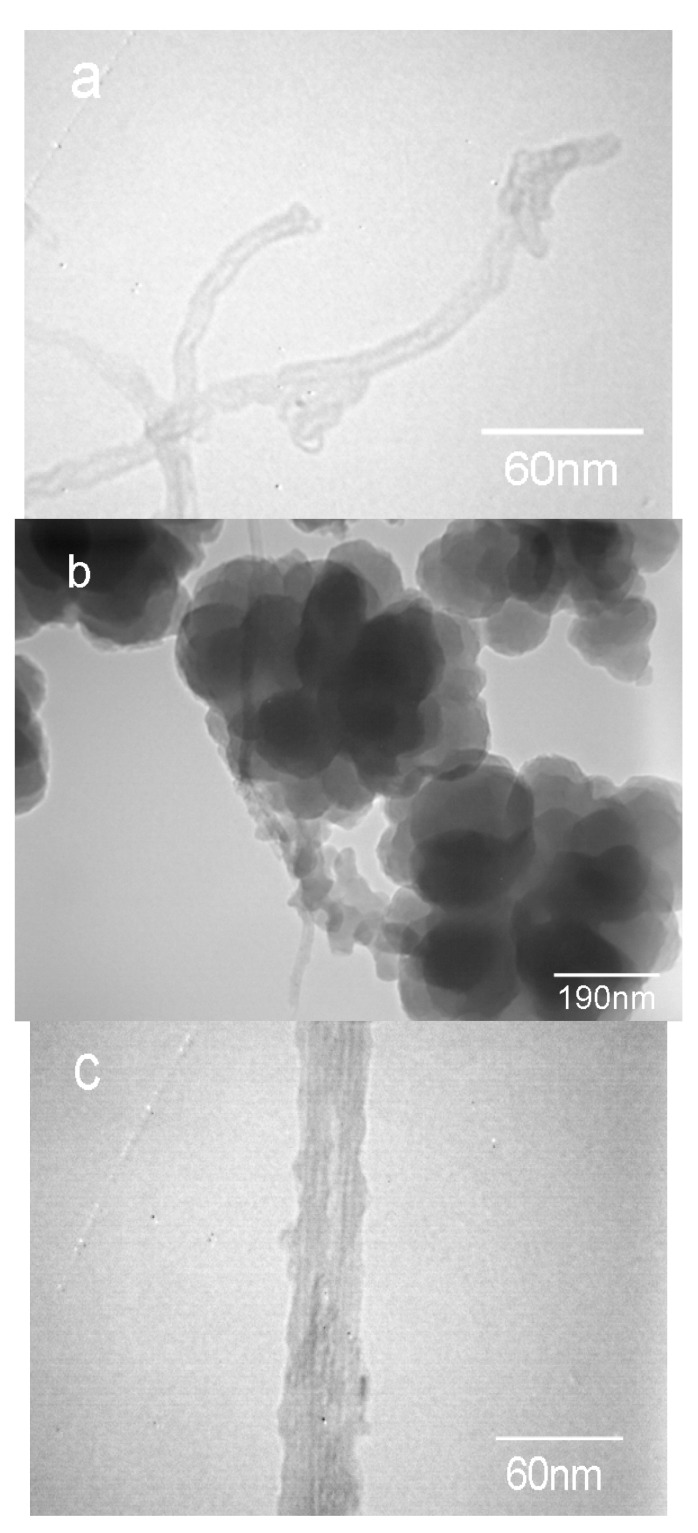
TEM images of (**a**) amino-CNTs, (**b**,**c**) amino-CNT/PAN microspheres with different magnifications, TEM: transmission electron microscopy; CNT: carbon nanotube; PAN: polyacrylonitrile.

**Figure 2:**
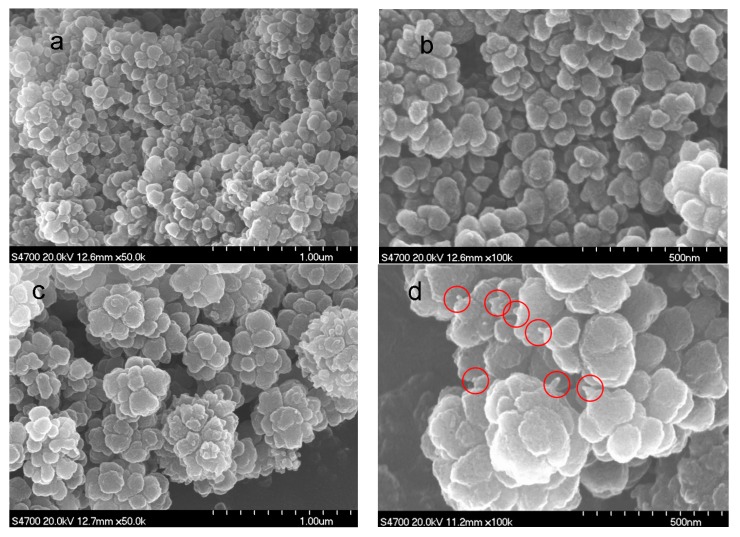
Scanning electron microscopy (SEM) images of (**a**,**b**) the PAN homopolymer and (**c**,**d**) the amino-CNT/PAN microspheres.

To investigate the morphology of amino-CNTs and the dispersion of amino-CNTs in the PAN matrix, TEM images of these samples are shown in Figure 1. The amino-CNTs with a diameter of 10~15 nm, as shown in Figure 1a, are individually dispersed after functionalization modification. Figure 1b illustrates that the amino-CNTs are embedded in the PAN matrix with a homogeneous dispersion. The TEM image of the individual amino-CNTs in the microspheres is individually shown in Figure 2c, with a diameter in the range of 25~35 nm, which is higher than that of amino-CNTs. Also, the outer surface of the amino-CNTs became rough, indicating that the PAN macromolecular chains were attached onto the surface of CNTs and formed a coating layer around the outer CNTs [25]. These results show that the microspheres with amino-CNTs exhibit homogeneous dispersion as well as good interfacial bonding between the PAN homopolymer matrix and the CNTs [9,26].

The morphologies of the PAN homopolymer and the amino-CNT/PAN microspheres were examined by SEM, as shown in Figure 2. It can be seen that the SEM images of the PAN homopolymer microspheres prepared by aqueous precipitation polymerization, as shown in Figure 2a,b, consist of numerous irregular balls with a diameter ranging from 50 to 100 nm. Compared with the PAN homopolymer microspheres, the effects of the amino-CNTs on the morphology of the amino-CNT/PAN microspheres can be clearly seen in Figure 2c,d, although the mass content of amino-CNTs in the microspheres is only 1%. The composite microspheres with a diameter about 300–400 nm after in situ polymerization consist of smaller nanoparticles with a diameter ranging from 70–200 nm. On the fringe of the microspheres in Figure 2d, there are some needle structures (shown in the ring) coming out from the outer surface of the microspheres, which are ascribed to the amino-CNTs [10]. The diameter of the amino-CNTs (about 30 nm) is larger than that of the amino-CNTs from the TEM images (Figure 1a); this difference is attributed to the PAN homopolymer grafting onto the outer surface of the amino-CNTs.

### 3.2. FTIR Spectra Analysis

**Figure 3:**
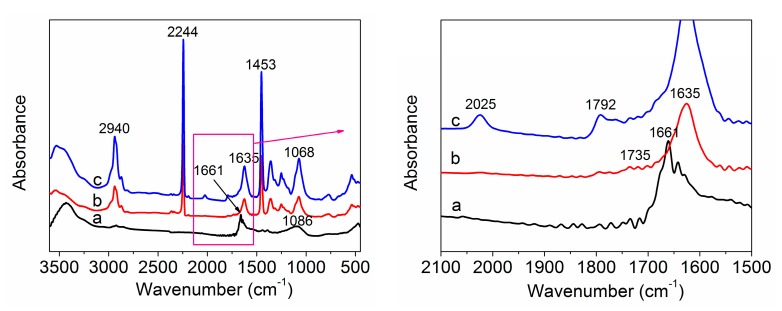
Fourier transform infrared (FTIR) spectra of (**a**) amino-CNTs, (**b**) PAN homopolymer and (**c**) amino-CNT/PAN microspheres.

The FTIR spectrum was used to analyze the chemical structure of samples. The FTIR spectra of the amino-CNTs, PAN homopolymer, and amino-CNT/PAN microspheres are shown in Figure 3. The FTIR spectrum of amino-CNTs, as shown in Figure 3a, shows an intensive absorption peak at 1661 cm^−1^, which is attributed to the stretching vibration of the amide carbonyl (C=O) groups. The peak at 1086 cm^−1^ is assigned to the stretching vibration of the C-N bond. These results indicate that the TETA molecules were covalently bonded to the surface of CNTs [27,28,29]. Figure 3b depicts the FTIR spectrum of the PAN homopolymer; the strong absorption peak at 2244 cm^−1^ is assigned to the vibration of the nitrile group, and the peak at 2940 cm^−1^ is attributed to the stretching vibration of the –CH_2_ groups. In Figure 3b, the relatively strong peak at 1635 cm^−1^ and the very weak peak at 1735 cm^−1^ are assigned to the hydrolysis of AN units during the polymerization [30]. The peaks at 1453 and 1068 cm^−1^ are assigned to bending vibration of the C–H groups. Compared with the PAN homopolymer, the spectrum of the amino-CNT/PAN microspheres is similar to that of the PAN, but there is a new peak appearing at 2025 cm^−1^ (as shown in Figure 3c), which is attributed to the chemical band (–C=C=N–) of reaction between the amino-CNT and PAN homopolymer macromolecular chains. Meanwhile, at 1792 cm^−1^ the microspheres show significant absorbance compared with the PAN, which indicates that the amino-CNTs influenced this group absorption. Thus, it is concluded that the amino groups of CNTs participated in the reaction and formed chemical bonds with the PAN during in situ polymerization.

### 3.3. XRD Curves

**Figure 4:**
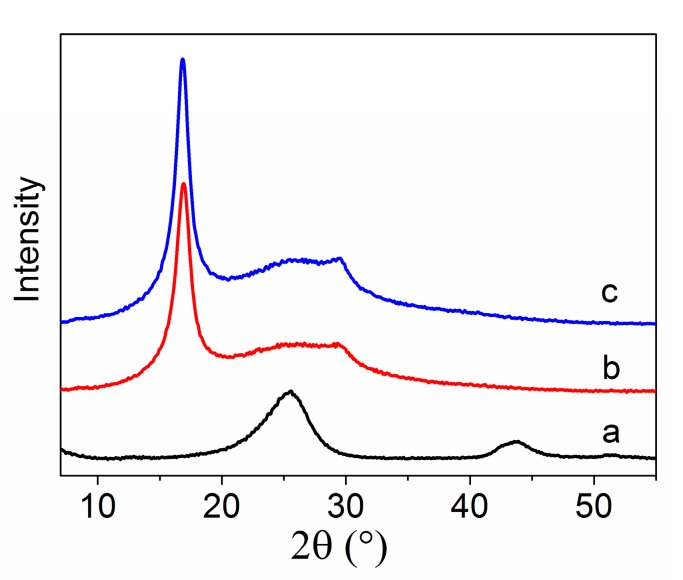
X-ray diffraction (XRD) patterns of (**a**) amino-CNTs, (**b**) PAN homopolymer and (**c**) amino-CNT/PAN microspheres.

XRD patterns of amino-CNTs, PAN homopolymer, and amino-CNT/PAN microspheres are shown in Figure 4. The XRD pattern of amino-CNTs, as shown in Figure 4a, shows a strong intensity at 25.6° and a weak intensity at 43.9°, which correspond to the (002) and (100) planes [31], respectively, of the graphite crystal structure. The XRD pattern of the PAN homopolymer in Figure 4b shows an intense peak (100) plane located at 2θ ≈ 17° and a weak peak (020) plane at 2θ ≈ 29°. The first intense peak at 2θ ≈ 17° is considered to be the reflection of the hexagonal lattice with a stiff rod-like conformation due to intermolecular repulsion of the nitrile dipoles. The second small peak at 2θ ≈ 29° corresponds to the second-order diffraction of the intense peak at 2θ ≈ 17° [32,33]. These results prove that the PAN homopolymer belongs to the semi-crystalline polymer category. The broad and weak peak at about 2θ ≈ 25° is ascribed to the amorphous structure of the PAN homopolymer [34]. The amino-CNT/PAN microspheres and the PAN homopolymer have similar XRD curves. However, the intensity of the (100) peak for the amino-CNT/PAN microspheres significantly improve compared to that of the PAN homopolymer, and the peak at about 2θ = 25° is more obvious than that of the PAN homopolymer due to the addition of amino-CNTs. The results from the XRD curves illustrate that the incorporation of amino-CNTs in the PAN matrix do not change the crystalline structure.

The crystallinity and crystal size of the PAN hompolymer and amino-CNT/PAN microspheres obtained from the XRD curves are shown in Table 1. The crystallinity of the PAN homopolymer calculated from Formula (2) is 35.05%, which is attributed to the (100) plane of the hexagonal lattice. The crystal size of the PAN homopolymer calculated from the Scherrer equation is 53.40 Å, which is ascribed to the stiff rod-like conformation due to the intermolecular repulsion of the nitrile dipoles. However, the crystallinity and the crystal size of the amino-CNT/PAN microspheres is 31.41% and 57.17 Å, respectively. The lower crystallinity of the amino-CNT/PAN microspheres compared to that of the PAN homopolymer is because the amino-CNTs disrupt the fairly regular macromolecular structure of the PAN homopolymer [35,36,37]. However, the higher crystal size of the microspheres is attributed to the nano-size of the CNTs, which induces the PAN macromolecular chain orientation around the surface of amino-CNT [38], similar to the CNT/PAN composite fibers [12,39].

### 3.4. Thermal Properties

**Figure 5:**
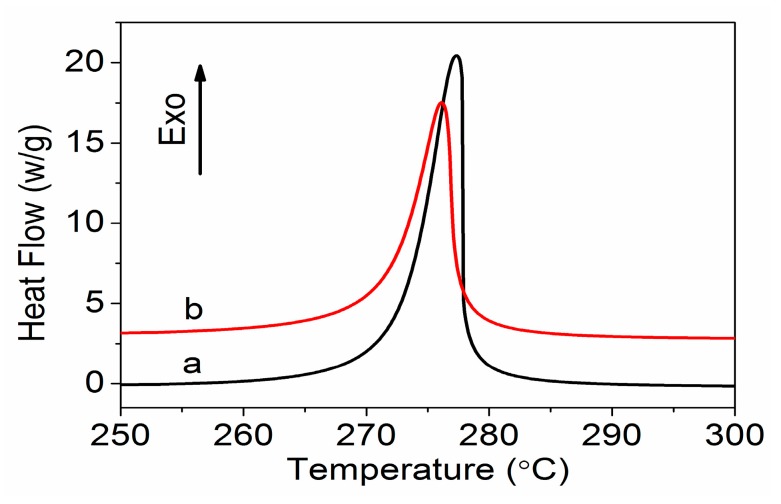
Differential scanning calorimetry (DSC) curves of (**a**) PAN homopolymer and (**b**) amino-CNT/PAN microspheres in a nitrogen atmosphere.

DSC thermograms of the PAN homopolymer and amino-CNT/PAN microspheres under a nitrogen atmosphere at a rate of 10 °C min^−1^ are shown in Figure 5. As only a cyclization reaction took place without oxidative reactions in the nitrogen atmosphere, there was one exothermic peak for the PAN homopolymer and amino-CNT/PAN microspheres. However, the exothermic peak of the amino-CNT/PAN microspheres had a lower intensity value than that of the PAN homopolymer. A lot of heat was released in these processing process. The initial temperature (*T_i_*), the final temperature (*T_f_*), their difference (*ΔT* = *T_f_* − *T_i_*), the peak temperature (*T_p_*), the evolved heat (*ΔH*), and the velocity of evolving heat (*ΔH*/*ΔT*) are all shown in Table 2.

From Table 2, we can see that the amino-CNT/PAN microspheres have different exothermic parameters compared to the control PAN homopolymer. The amino-CNT/PAN microspheres have a lower initial temperature compared with the PAN hopolymer, which may be attributed to the new chemical bond between the amino-CNTs and PAN macromolecular chains inducing the onset of the cyclization reaction. Moreover, the peak temperature of samples shifted from 277.3 °C for the PAN homopolymer to 276.2 °C for the amino-CNT/PAN microspheres. The final temperature and the evolved heat are basically unchanged. Furthermore, the amino-CNT/PAN microspheres have a larger *ΔT* and a smaller *ΔH*/*ΔT* compared with the PAN matrix, indicating that the heat release was not concentrative and expeditious. These results suggest that the addition of amino-CNTs was conducive to the reaction of cyclization and controlled the exothermic reaction.

**Figure 6:**
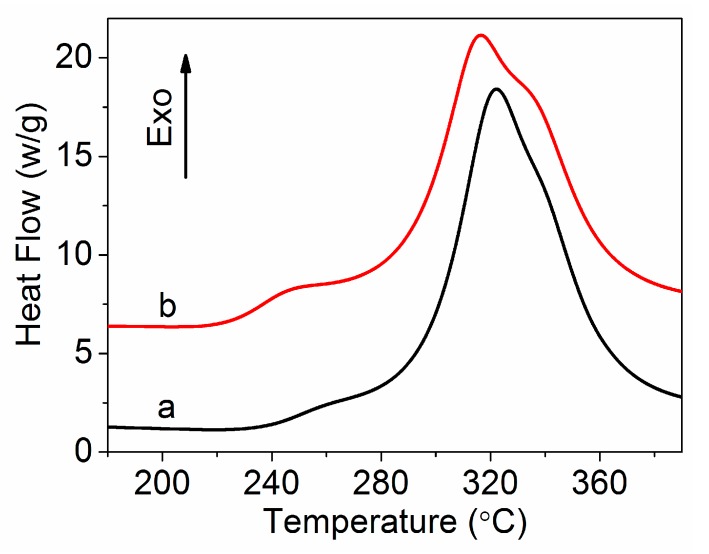
DSC curves of (**a**) PAN homopolymer and (**b**) amino-CNT/PAN microspheres in air atmosphere.

DSC thermograms of the PAN homopolymer and amino-CNT/PAN microspheres under air atmosphere at a rate of 10 °C min^−1^ are illustrated in Figure 6. The curves of the DSC in the air atmosphere have two exothermic peaks, which is different from the curves of the DSC in the nitrogen atmosphere that had one intensive exothermic peak. The first exothermic peak is assigned to the cyclization reactions and the second exothermic peak is attributed to the oxidative reactions. The relative intensity of the heat flow for the amino-CNT/PAN microspheres is lower than that of the control PAN homopolymer. The initial temperature (*T_i_*), the final temperature (*T_f_*), their difference (*ΔT* = *T_f_* − *T_i_*), the peak temperature (*T_p_*), the evolved heat (*ΔH*), and the velocity of evolving heat (*ΔH*/*ΔT*) are all shown in Table 3.

As shown in Table 3, the *T_i_* of the PAN homopolymer is 238.1 °C. However, the addition of amino-CNTs into the PAN polymer decreases the *T_i_* from 238.1 to 224.2 °C compared with the PAN polymer, which indicates that the amino-CNT/PAN microspheres participate in chemical reactions at the lower temperature. The *T_p_* of the amino-CNT/PAN microspheres also shifted from 322.1 to 316.7 °C compared with the control PAN homopolymer. However, the value of ΔH remained basically the same. The microspheres with broader *ΔT* and lower *ΔH*/*ΔT* suggest that the heat release was more easily controlled by the microspheres than by the PAN homopolymer. These results indicate that the addition of amino-CNTs in the PAN homopolymer facilitated the exothermic reaction in the air atmosphere.

**Figure 7:**
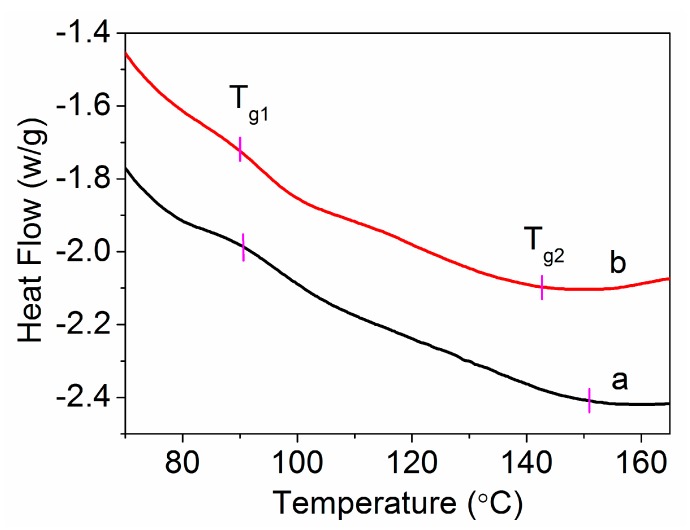
The *T_g_* of (**a**) PAN homopolymer and (**b**) amino-CNT/PAN microspheres.

The glass transition temperature (*T_g_*) of the PAN homolymer and amino-CNT/PAN microspheres with at a heating rate of 60 °C min^−1^ is shown in Figure 7. The samples were obtained by aqueous phase precipitation in situ polymerization, so they were belonged to the unorientation of polymer. Bshair et al. [36,40] regarded that the unoriented PAN polymer had two glass transitions and the oriented PAN polymer only had one glass transition. The lower glass transition temperature (*T_g_*_1_) is ascribed to the molecular motion of the cyano orientation, and the higher glass transition temperature (*T_g_*_2_) is attributed to the amorphous regions. For the PAN homopolymer, as shown in Figure 7, the glass transition temperatures are 90.6 and 151.0 °C, respectively. The addition of amino-CNTs into the PAN matrix decreased the glass transition temperature. The *T_g_*_1_ decreased from 90.6 to 89.8 °C, which was assigned to the reaction between amino-CNTs and cyano on the PAN macromolecular structure that weakened the interaction between cyano and cyano. So, the molecular motion of amino-CNT/PAN microspheres moved more easily than that of the PAN homopolymer. The *T_g_*_2_ decreased from 151.0 to 142.5 °C, which was attributed to the amino-CNT/PAN microspheres having lower crystallinity. Kong et al. [41,42] reported that the degree of crystallinity could be measured by DSC curves. So, the lower *T_g_* of amino-CNT/PAN microspheres might be assigned to the lower degree off crystallinity. These were also confirmed by the XRD results.

## 4. Conclusions

In this study, amino-CNT/PAN microspheres were obtained by precipitation in situ polymerization under aqueous solvent. The morphology of the amino-CNT/PAN microspheres with a diameter of 300–400 nm, observed by SEM and TEM images, showed that amino-CNTs were homogenously dispersed in the PAN homopolymer matrix and that they enhanced the interfacial bonding between the amino-CNTs and the PAN matrix. The results from the FTIR spectra also confirmed that the amino groups of CNTs participated in the chemical reaction and formed a new chemical bond with the PAN homopolymer during in situ polymerization. The XRD analysis showed that the amino-CNT/PAN microspheres had lower crystallinity than the control PAN homopolymer due to the amino-CNT interrupting the regular arrangement of cyano groups in PAN macromolecular chains and forming new chemical bonds. The orientation of the PAN chain around the surface of the amino-CNTs resulted in a large crystal size of amino-CNT/PAN microspheres. The amino-CNT/PAN microspheres had a lower initial temperature and maximum temperature compared to the PAN matrix in a nitrogen atmosphere or air atmosphere, and the relative intensity of the exothermic maximum peak of the amino-CNT/PAN microspheres was lower than that of the PAN homopolymer. The addition of amino-CNTs into the PAN matrix had broader *ΔT* and lower *ΔH*/*ΔT* than the control PAN homopolymer, and it decreased the glass transition temperature of the PAN homopolymer.

## Figures and Tables

**Table 1:** XRD data of PAN homopolymer and amino-CNT/PAN microspheres, XRD: X-ray diffraction; CNT: carbon nanotube; PAN: polyacrylonitrile.

Sample	Crystallinity (%)	Crystal Size (Å)
PAN	35.05	53.40
Amino-CNT/PAN	31.41	57.17

**Table 2:** Differential scanning calorimetry (DSC) data for PAN homopolymer and amino-CNT/PAN microspheres in a nitrogen atmosphere.

Sample	*T_i_* (°C)	*T_p_* (°C)	*T_f_* (°C)	*ΔH* (J g^−1^)	*ΔT* (°C)	*ΔH*/*ΔT* (J g^−1^ °C^−1^)
PAN	267.0	277.3	281.5	567.1	14.5	39.1
Amino-CNT/PAN	263.9	276.2	281.4	565.7	17.5	32.3

**Table 3:** DSC data for PAN homopolymer and amino-CNT/PAN microspheres in air atmosphere.

Sample	*T_i_* (°C)	*T_p_* (°C)	*T_f_* (°C)	*ΔH* (J g^−1^)	*ΔT* (°C)	*ΔH*/*ΔT* (J g^−1^ °C^−1^)
PAN	238.1	322.1	375.2	4738	137.1	34.6
Amino-CNT/PAN	224.2	316.7	375.3	4758	151.1	31.5
